# Behavioral tuning of spider silk thread stiffness circumvents biomaterial trade-offs

**DOI:** 10.1073/pnas.2529200123

**Published:** 2026-01-26

**Authors:** Jonas O. Wolff, Daniela C. Rößler, Anna-Christin Joel, Vincent Jackel, Sebastian Büsse, Peter Michalik, Martín J. Ramírez

**Affiliations:** ^a^Evolutionary Biomechanics, Zoological Institute and Museum, University of Greifswald, Greifswald 17489, Germany; ^b^School of Natural Sciences, Faculty of Science and Engineering, Macquarie University, Sydney, NSW 2109, Australia; ^c^Abteilung II - Biodiversität der Tiere, Bonner Institut für Organismische Biologie, University of Bonn, Bonn 53121, Germany; ^d^Spider Silk lab, Institute of Biology II, Rheinisch-Westfälische Technische Hochschule Aachen University, Aachen 52074, Germany; ^e^Cytology and Evolutionary Biology, Zoological Institute and Museum, University of Greifswald, Greifswald 17489, Germany; ^f^Zoological Museum, Zoological Institute and Museum, University of Greifswald, Greifswald 17489, Germany; ^g^Division of Arachnology, Museo Argentino de Ciencias Naturales “Bernardino Rivadavia”, Consejo Nacional de Investigaciones Científicas y Técnicas, Buenos Aires 1405, Argentina

**Keywords:** hybrid material, metastructure, spider web, mechanical trade-off, adaptive material

## Abstract

Biological polymers often face a trade-off between stiffness, strength, and extensibility: Materials that are strong and stiff tend to be brittle, while those that are elastic and extensible usually lack strength. Here, we show that netcasting spiders (Deinopidae) overcome this trade-off by forming mixed-silk metastructures, which enable both high elastic deformation and load resistance. These spiders have evolved a unique predatory strategy, casting a sticky silk web over prey, which subjects the web radii to extreme strains far exceeding those sustained by typical spider silk fibers. The radii consist of a compound filament with an elastomeric core surrounded by looped bundles of thin fibers. This architecture results in an unusual mechanical profile: The threads are initially compliant and highly extensible, but they stiffen as the fiber loops straighten, enhancing load-bearing capacity. Notably, spiders control this compound architecture through a reel-spinning technique, controlling loop formation and fiber mixture to establish an elasticity gradient across the web—stiff and strong in the main frame lines, yet soft and hyperelastic in the lower radii that undergo the greatest deformation during prey capture. These findings represent a unique case of behavioral modulation of silk processing to circumvent biomaterial trade-offs, enabling extraordinary dynamics and specialization of web architecture. The herein described principle of looped fiber-reinforced elastomers may also be transferred to the design of artificial materials for applications that require both high elasticity and strength.

Polymer materials have an inherent trade-off between high strength (or stiffness) and extensibility (or elasticity), as enhancing intermolecular interactions or chain rigidity improves load-bearing capability at the expense of chain mobility necessary for large deformations (1–3). Spiders that produce silk materials may overcome such limitations by behaviorally postprocessing silk fibers into metastructured composite materials. For instance, through special felting or combing techniques, certain spiders produce hyperextensible multifiber threads that incorporate high hidden lengths via regularly arranged hierarchical loop patterns with a controlled unraveling dynamics under tension (4, 5). Orb-web spiders address the conflict between elasticity and load-bearing capacity by suspending elastomeric lines within a scaffold of stiffer radial lines, resulting in efficient impact-absorbing structures (6–8). Nevertheless, there are instances of spider webs that undergo extreme, yet reversible, deformation during prey capture, which cannot be explained by any of the aforementioned principles.

In this study, we present a metastructure effect observed in the web of the enigmatic netcasters (Deinopidae). These spiders have evolved a distinctive predatory behavior, wherein the capture net is held with the anterior legs and propelled towards the prey (9, 10). During this dynamic movement, the web undergoes significant yet reversible deformations. While the capture lines of netcasters, known as cribellar threads, are recognized for their high extensibility, capable of sustaining elongations of over 150%, the major ampullate silk, which constitutes the web radii in orb-weavers, can only deform reversibly over a strain of 2 to 5% and fractures at elongations over 20% (3, 8, 10)—a threshold that is significantly surpassed during the netcaster´s strike. Conversely, silk lines with higher extensibilities may lack the capacity to support substantial prey masses due to the inherent trade-off between elasticity and load-bearing capacity. To elucidate the mechanisms enabling this unusual web dynamics, we investigated the properties, structure, and behavioral spinning of netcaster silk threads.

## Results

During the predatory strike, the central web area extended 8 to 24 times within 70 to 126 ms (N = 4), with the interior lower radius reaching strains of 90 to 200% (Movie S1). The outer upper, median, and lower radii experienced strains of 16%, 24%, and 39%, respectively (N = 1, Movie S2). The downward strike of the spider, initiated by launching towards prey, elastically deformed the frame. The immediate release of stored elastic energy facilitated the spider’s return to its initial position. As the dragline from which the spider is hanging from is comparably stiff (see below) and would impede this movement, it is initially maintained in a slack state. Its unraveling halts the downward movement, preventing the frame from overloading.

**Fig. 1.**
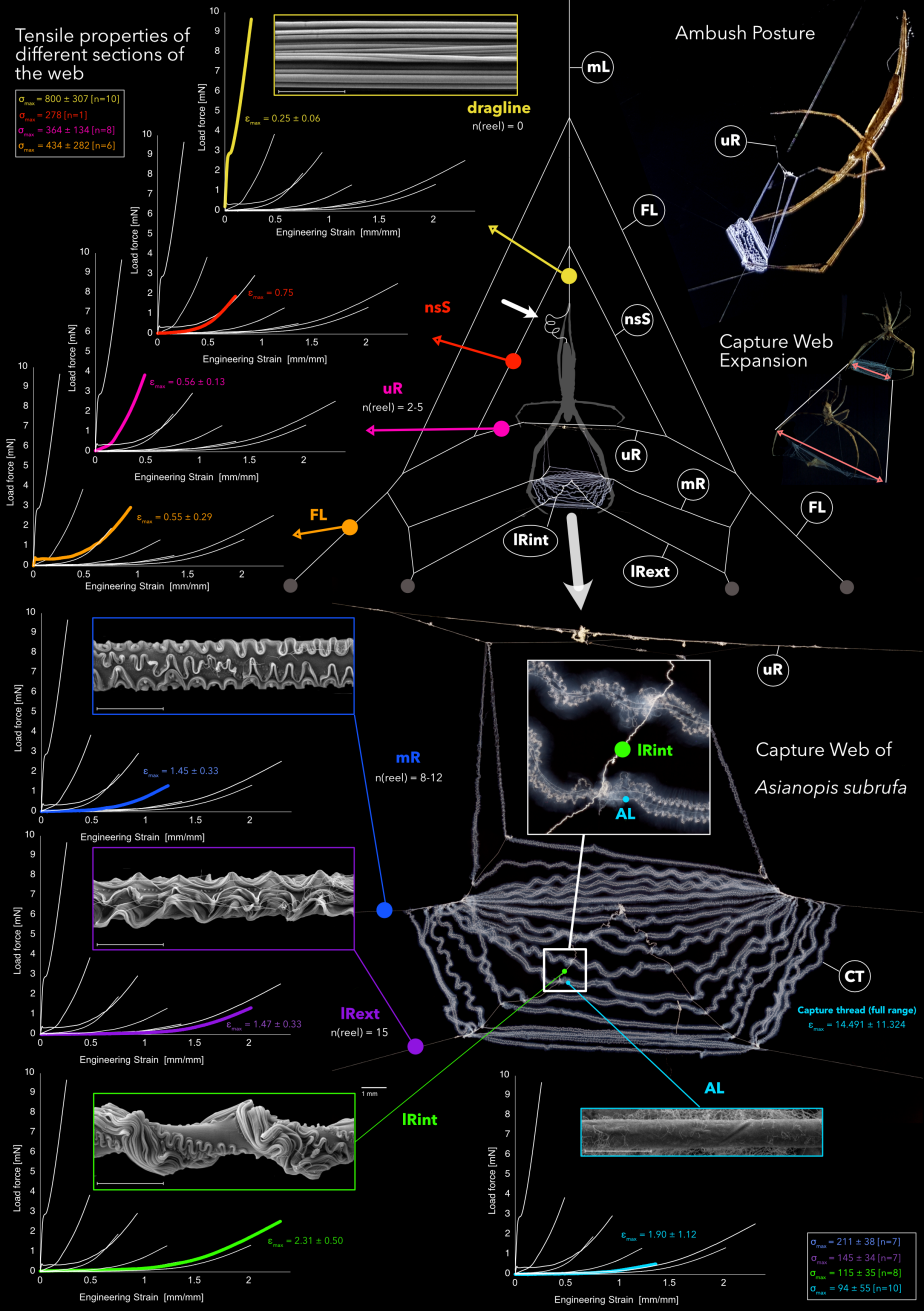
Web architecture of *Asianopis subrufa* (Deinopidae), with ultrastructure (Scale bar, 5 µm) and tensile properties (load force and strain) of different lines in the web. The spiders create a vertical stiffness gradient along the web by thread meta-structure tuning to circumvent trade-offs between silk elasticity and strength, and enable web hyperextension. Mechanical values given as mean and SD. ε_max_, ultimate engineering strain (extensibility) [mm/mm]; σ_max_, ultimate engineering strength [MPa]; n, number of samples tested; n(reel), number of reeling cycles (observed range, from three recorded events of web building). Line types: AL, axial line of capture thread; FL, frame line; lRext, lower radius—exterior; lRint, lower radius—interior; mR, median radius; nsS, nonsticky spiral; uR, upper radius.

The observed web deformation is attributable to the distinctive structure of the netcasters’ radii: Loops of thin fibers attach to a soft, highly elastic line (Fig. 1). To spin the radii, the spider initially attaches the silk to the lateral frame line. Then, it moves to the web hub, but before attaching the line there, it adds length by pulling the thread with its hind legs (Movie S3). During this reeling process, the posterior spinnerets show flickering movements, which may enhance loop formation of silk fibers added from these spinnerets (Movie S4). Notably, during silk reeling, the line was consistently observed to remain taut, indicating that it contracts after spinning.

The spiders modulate the extensibility of each radius and the frame elements by adjusting reeling cycles and thread composition, creating an elasticity gradient from stiff and strong in the main load bearing line (the dragline the spider hangs on; mean Young’s modulus 10.88 GPa) to initially soft and hyperelastic in the lower radius (mean Young’s modulus 0.01 GPa; ultimate strain ~10 times of dragline at a third load at fracture; elastic strain ~100 times that of dragline) and axial line of cribellar capture threads (Fig. 1). The chimeric radii showed higher toughness (uR: mean 71.0 MPa; mR: mean 86.9 MPa; lRint: mean 75.1 MPa) than the axial line of the capture thread (mean 39.6 MPa).

The web usually experiences multiple events of extreme expansions over its lifetime (Movie S5). Cyclic loading tests on the lower radius revealed extreme hysteresis and stress relaxation at maintained strain (dσ = 16 MPa), indicating high viscoelasticity. Subjecting the lower radius to repeated extreme (150%) strain did not diminish its elasticity but affected its load-bearing capacity (between cycle 1 and 2 1.8% stress loss, between following cycles 12.3 and 21.3% stress loss, but recovery of 8.4% stress between cycles 6 and 7).

## Discussion

Herein, we present a silk metastructure principle—a chimeric looped fiber-reinforced elastomer—that integrates both elasticity and strength. Upon extension, these composite threads are initially very soft, facilitating prolonged and effortless stretching. Subsequently, the looped fiber bundles straighten, thereby imparting considerable strength and preventing the thread from breaking under the load of captured prey. The structural configuration likely arises from the postspin contraction of the elastomeric core, which induces coiling of the adjoined fibers. This effect could be replicated in artificial fiber materials by affixing stiff micro- or nanofibers to strained elastomeric materials, resulting in the formation of loops upon elastomer relaxation. This approach offers promising avenues for material design.

Significantly, the looped fiber-reinforced elastomer principle facilitates the customization of thread stiffness in various sections of the web, thereby enabling the integration of both web hyperextension and load-bearing capacity. Thread stiffness adjustments have been documented in orb-weaving spiders, which may modify radial prestrain to fine-tune sonic properties of distinct web regions (11). In this context, Young’s modulus, an indicator of stiffness, can be modified by an order of magnitude. Conversely, employing the looped fiber-reinforced elastomer principle, netcasters can adjust thread stiffness across a range of up to three orders of magnitude by regulating the degree of coiling of the adjoined fibers. Coiled fiber metastructures have recently been highlighted in silk materials as a toughness-enhancing mechanism (4, 5); however, thus far, only with irreversible deformation. In contrast, the looped fiber-reinforced elastomers principle of netcaster web radii demonstrates a fiber metastructure that remains elastic throughout most of its extension and possesses adjustable stiffness.

The elastomeric core fiber diameter and initial Young’s modulus of the netcaster radii match the axial fibers of the cribellar threads, indicating the same silk material (pseudoflagelliform silk). The frame lines, however, show high initial stiffness but follow the elastic, strain-hardening behavior of the upper radius, suggesting a mix of ampullate and pseudoflagelliform silks. Further studies on glandular origin and viscoelastic properties are needed to understand deinopid web biomechanics.

## Materials and Methods

High-speed videos of predatory strikes were recorded with 1,000 to 1,300 fps. Dynamic strains were calculated as the maximal observed length divided by the initial length of lines. Constructing behavior was filmed overnight under (infra-)red light.

Silk threads were sampled from webs and abseiling spiders. Mechanical properties were measured with quasistatic tensile tests (12). The thread structure was studied with polarized light microscopy and field emission scanning electron microscopy.

Additional methodology details are provided in *SI Appendix*.

## Supplementary Material

Appendix 01 (PDF)

Dataset S01 (CSV)

Movie S1. Predatory strike of *Asianopis subrufa* showing web deformation, captured in captivity inside a glass terrarium (recorded with 1,300 frames per second, vertically, from below the terrarium, under white light, contrast enhanced).

Movie S2. Predatory strike of *Asianopis subrufa* showing web deformation, captured in captivity inside a plastic cup (recorded with 1,300 frames per second, horizontally, under white light, contrast enhanced).

Movie S3. Sequence of web radius construction (upper, median and lower radius) in *Asianopis subrufa*, captured in captivity inside a glass terrarium (recorded horizontally under red light).

Movie S4. Detail of web radius construction in *Asianopis subrufa*, showing the leg and spinneret movements, captured in captivity inside a glass terrarium (recorded vertically, from below the terrarium, under infrared light).

Movie S5. Detail of web expansion by an ambushing *Asianopis* sp. from a sequence recorded in the field. The second half of the video shows the same extension at 10% playback speed. This behavior was observed directly after web building was completed and the ambush posture was adopted, but also when staying in the ambush posture for a prolonged time (here: 6.5 hours after web construction). It may have the function to test or modulate web elasticity.

## Data Availability

All study data are included in the article and/or supporting information.
