# Supplementary material for: Behavioral tuning of spider silk thread stiffness circumvents biomaterial trade-offs
Source: Proc Natl Acad Sci U S A. 2026 Jan 26;123(5):e2529200123. doi: 10.1073/pnas.2529200123 (PMC12867701; doi:10.1073/pnas.2529200123)
Supplement: Supplementary file 1 — Appendix 01 (PDF) [file pnas.2529200123.sapp.pdf]

PNAS

Supporting Information for
Behavioral tuning of spider silk thread stiffness circumvents biomaterial trade-offs.

Jonas O. Wolff*, Daniela C. Rößler, Anna-Christin Joel, Vincent Jackel, Sebastian Büsse, Peter Michalik, and Martín J. Ramírez.

* corresponding author: Jonas O. Wolff.
Email: j.wolff@uni-greifswald.de

This PDF file includes:

Supporting text
Legends for Movies S1 to S5
Legend for Dataset S1
SI References

Other supporting materials for this manuscript include the following:

Movies S1 to S5
Dataset S1

Supporting Information Text

Extended Material and Methods

Spider sourcing and maintenance. For the biomechanical and microscopy study 15 juvenile (2 male, 2 female, remainder undetermined sex) Australian netcasters (*Asianopis subrufa*) were collected in Bidjigal Reserve, West Pennant Hills, Australia, and brought to the lab, where they were kept in inverted plastic cups (bottom diameter 12 cm, top diameter 9 cm, height 13 cm) in a temperature-controlled room (day 8h 26°C, night 12h 20°C, 2h transition morning and evening, relative humidity 70%). Spiders were sprayed with water twice a week and fed with one cricket (*Acheta domesticus*) per week. Two matured female spiders, after molting, were transferred to glass terrariums (20×20×40 cm) for better accessibility of the webs for sampling and for better visibility of the web mechanics during high-speed video recordings.

For the observation of web building in Video S3, 3 juvenile *Asianopis subrufa* (~2 cm body length, females and males) were collected in public parks and on the campus in Sydney (export permit number: PWS2019-AU-000248). Spiders were kept individually in clear plastic containers (8 x 6 x 6 cm, Jousi boxes) with tooth picks as climbing support and black cardboard as background. Their container was kept moist with the help of wetted cotton balls. *Acheta domesticus* or *Lucilia sericata* was fed twice per week.

For the observation of web building behavior in Video S4, 5 *Asianopis subrufa* females were purchased from a captive breeding program in Australia (Minibeast, export permit number: PWS2023-AU001805). Spiders were kept in clear plastic containers (10 x 10 x 18 cm) filled with a wooden backwall and twigs under a 12:12 light cycle in a temperature-controlled room (day 25-26°C, night 22°C, relative humidity in the boxes 70-80%). Spider were fed *ad libitum* with crickets and the boxes sprayed with water twice a week.

Additional observations of *Asianopis* sp. were made in the field in Diwan, Queensland, Australia, for which an infrared camera (full spectrum modified Nikon Z6 with a 50mm/2.8 lens, filming at 60 frames per second) was installed to monitor the activity of the spider throughout the night, including web building, web handling and prey capture. Field observations confirmed that the behavior observed in the lab was similar to the behavior performed in the natural habitat. Video S5 shows an expansion of the web from a sequence filmed during these observations.

Filming of prey capture strikes. High-speed video recordings of the capture strikes were done with a post-triggered Phantom MIRO LC 320S camera equipped with a Canon 100mm macro lens, filming at 1,000-1,300 fps from below the container, illuminated with two flashlights (Heider CFX Super Power, Heider AGX Ultra Power). Supplementary, three synchronized Sony DSC-RX0 II Cameras (Sony Corporation) modified with a Back-Bone Ribcage RX0-II MFT-mount adapter (Back-Bone Gear Inc.), equipped with Samyang 100mm/2.8 macro lenses and synchronized with Sony CCB-WD1 controllers, were used. Here, we recorded at 1,000 fps with pre-trigger from two lateral angles and from below. For this purpose, two opposite sides of the plastic cup were cut open, and the inverted cup (with the spider and its web in it) was placed on a transparent Perspex sheet. In two cases, the spiders were filmed in a glass terrarium (see above).

The dynamic changes in the central web area and the thread lengths within was measured in video stills of the recordings taken from below, using ImageJ (1), considering the initial area or length (before strike) and the maximal observed extension. Changes in outer radii length were measured from video stills of a lateral video recording. Strains were calculated as the maximal observed length (in pixels) divided by the initial length. We note that, while we took these measurements from the most planar angle, the determined dynamic strain values represent a rough estimate, as the motion takes place in an three-dimensional space and perspective distortion could not be fully mitigated by our approach.

Filming of construction behavior. Construction behavior in Video S3 was filmed using a webcam (Logitech C920 HD Pro) and the matching software “LogiCapture” with 30 fps. Red led light were used to illuminate the setting. The spiders’ behavior was filmed the complete night.

Constructing behavior in Video S4 was filmed using a full spectrum modified DSLR camera (Nikon D7200) with a macro lens (Nikon AF-S Micro NIKKOR 60mm F2.8G ED) and an external IR light source (48 LED CCTV light, CENPEK).

Biomechanical tests. For the pointed measurements of each web element, webs were collected on custom-cut cardboard frames lined with double-sided tape. These whole-web mounts were then investigated under a dissection microscope, and clean sections were identified for each line type. These sections were fixed onto cardboard strips, attached to the rectangular cardboard frame of the whole-web mount and with a terminal C-shaped notch (gauge length 6-12 mm) lined with double-sided adhesive tape, catching the targeted web section. After fixing different web sections this way, the line samples were carefully cut free, using a soldering iron with a fine, pointed tip. Draglines were collected by letting the spider lower themselves from a paintbrush. The resulting drop-down dragline was then collected above the hanging spider with cardboard frames (window size 11 mm) lined with double-sided adhesive tape.
All samples (Dataset S1) were stored in dry boxes, away from sunlight, in an air-conditioned room at 23-25°C and 25-65% relative humidity, the same conditions under which tensile tests were performed.
Before tensile testing, the silk line samples were fixed to the edges of their cardboard holders with Elmer’s glue and any excessive cardboard was removed. Each prepared sample was checked with an inverted polarized transmission light microscope (AXIO Vert. A1, Carl Zeiss, Jena, Germany) under an EC EPN 100×/0.9 lens, and photographed with an Axiocam 208 color camera with the ZEN 3.7 software. From these photos in the ZEN software, the diameter of the lines was measured (whole bundle, at its narrowest section, with all fibers included, except for the cribellar threads, where only the width of the axial line was measured), following the approach from Blackledge et al. (2). The sample was then mounted with its C-shaped cardboard holder into a T150 Universal Testing Machine (KLA, Milpitas, California, USA) using clamps. Force-extension curves were recorded with a strain rate of 1%, representing a quasi-static test to exclude viscoelastic effects (2, 3). Tests were run until fracture of the sample. For the capture threads, the first fracture (drop in force) was taken as indication that the axial line broke. Engineering stress was calculated by dividing the load force values by the cross-sectional area of the bundle in its narrowest point (based on diameter measurements, assuming a cylindrical architecture and close fiber packing). We also performed a stress relaxation test in which a lower radius sample was strained to 150%, held for 30.5s and then relaxed back to 0% strain. This was repeated seven times, with a rest time of 3 min in between, to observe energy losses and recovery of tensile properties.

Scanning Electron Microscopy. For the electron microscopy study, the silk samples were collected as whole-web mounts on custom cardboard frames, as mentioned above and subsampled with C-shaped cardboard pieces and then placed over glass microscopy slides. Some samples were taken and observed suspended from the glass background with silk samplers (4). Samples were coated for 120-180 seconds with AuPd with a sputter coater Quorum Technologies SC7620 and examined with a field-emission Zeiss GeminiSEM360 under high vacuum.

Movie S1 (separate file). Predatory strike of *Asianopis subrufa* showing web deformation, captured in captivity inside a glass terrarium (recorded with 1,300 frames per second, vertically, from below the terrarium, under white light, contrast enhanced).

Movie S2 (separate file). Predatory strike of *Asianopis subrufa* showing web deformation, captured in captivity inside a plastic cup (recorded with 1,300 frames per second, horizontally, under white light, contrast enhanced).

Movie S3 (separate file). Sequence of web radius construction (upper, median and lower radius) in *Asianopis subrufa*, captured in captivity inside a glass terrarium (recorded horizontally under red light).

Movie S4 (separate file). Detail of web radius construction in *Asianopis subrufa*, showing the leg and spinneret movements, captured in captivity inside a glass terrarium (recorded vertically, from below the terrarium, under infrared light).

Movie S5 (separate file). Detail of web expansion by an ambushing *Asianopis* sp. from a sequence recorded in the field. The second half of the video shows the same extension at 10% playback speed. This behavior was observed directly after web building was completed and the ambush posture was adopted, but also when staying in the ambush posture for a prolonged time (here: 6.5 hours after web construction). It may have the function to test or modulate web elasticity.

Dataset S1 (separate file). Thread samples and results of individual tensile tests.

SI References

1. C. A. Schneider, W. S. Rasband, K. W. Eliceiri, NIH Image to ImageJ: 25 years of image analysis. *Nat methods* **9**, 671-675 (2012).

2. T. A. Blackledge, R. A. Cardullo, C. Y. Hayashi, Polarized light microscopy, variability in spider silk diameters, and the mechanical characterization of spider silk. *Invertebrate Biology* **124**, 165-173 (2005).

3. G. Greco, B. Schmuck, S. Jalali, N. M. Pugno, A. Rising, Influence of experimental methods on the mechanical properties of silk fibers: A systematic literature review and future road map. *Biophysics Reviews* **4** (2023).

4. M. J. Ramirez, A. M. Ravelo, L. Lopardo, A simple device to collect, store and study samples of two-dimensional spider webs. *Zootaxa* **3750**, 189-192 (2013).
